# Lifestyle Intervention Improves Metabolic Dysfunction-Associated Steatotic Liver Disease in Children with Down Syndrome

**DOI:** 10.3390/nu17142331

**Published:** 2025-07-16

**Authors:** Vittorio Scoppola, Annalisa Crudele, Antonella Mosca, Nadia Panera, Chiara di Camillo, Caterina Bock, Massimiliano Raponi, Alberto Villani, Anna Alisi, Diletta Valentini

**Affiliations:** 1 Research Unit of Genetics of Complex Phenotypes, Bambino Gesù Children’s Hospital, IRCCS, 00165 Rome, Italy; vittorio.scoppola@opbg.net (V.S.); annalisa.crudele@gmail.com (A.C.); nadia.panera@opbg.net (N.P.); 2 Hepatology and Liver Transplant Unit, Bambino Gesù Children’s Hospital, IRCCS, 00165 Rome, Italy; antonella.mosca@opbg.net; 3 General Pediatrics and ED II Level Unit, Bambino Gesù Children’s Hospital, IRCCS, 00165 Rome, Italy; chiara.dicamillo@opbg.net (C.d.C.); alberto.villani@opbg.net (A.V.); 4 Diagnostic and Interventional Radiology Unit, Bambino Gesù Children’s Hospital, IRCCS, 00165 Rome, Italy; caterina.bock@opbg.net; 5 Medical Direction, Bambino Gesù Children’s Hospital, IRCCS, 00165 Rome, Italy; massimiliano.raponi@opbg.net; 6 General Pediatric Folllow-Up Unit, Bambino Gesù Children’s Hospital, IRCCS, 00165 Rome, Italy; diletta.valentini@opbg.net

**Keywords:** down syndrome, MASLD, antioxidants, mediterranean diet, cytokines

## Abstract

**Background/Objectives**: We evaluated the efficacy of a good lifestyle intervention on the severity of metabolic dysfunction-associated steatotic liver disease (MASLD) in children with Down syndrome (DS). **Methods**: This retrospective longitudinal study included 31 children with Down syndrome (DS) who were affected by MASLD and attended nutritional counseling based on a nutritional approach (e.g., Mediterranean diet and antioxidant supplements), as well as physical exercise. Clinical parameters, markers of low-grade systemic inflammation, and hepatic steatosis, as assessed by ultrasound, were evaluated at baseline (T0) and after 6 months (T1). **Results**: Several anthropometric and biochemical parameters, including body mass index, waist circumference, diastolic and systolic blood pressure, aspartate aminotransferase, basal insulin, insulin resistance, pro-inflammatory interleukin-1β, and anti-inflammatory interleukin-10, showed significant improvement after 6 months of a nutritional approach. This study also found a regression of at least one grade of hepatic steatosis in a significant portion of patients, especially in those who received antioxidant supplements. **Conclusions**: Our study further supports the hypothesis that a healthy lifestyle intervention, based on adherence to the Mediterranean diet, natural supplements with antioxidant properties, and regular physical activity, can be considered a safe therapeutic approach for reducing the risk and severity of MASLD in children with DS.

## 1. Introduction

Down syndrome (DS) represents the most common chromosomal alteration at birth [1] and is caused by the presence of an extra chromosome 21 in the genetic make-up. Although few studies have been conducted on the chronic health problems of people with DS, it is known that the risk of mortality from cardiovascular and metabolic diseases is higher in individuals with DS than in the general population [2].

Among the various medical conditions that affect children with DS, the most common is obesity [3]. The risk of obesity in children and adolescents with DS is higher than in the general population [4], due to constitutional, behavioral, and metabolic problems. In particular, the prevalence of being overweight and obesity in children with DS was 13.3–52.9% and 0–62.5%, respectively, compared to 20–25% being overweight and 9–10% obesity in the general pediatric population. Like the general population, obesity in children with DS may be associated with insulin resistance, dyslipidemia, cardiovascular disease, and obstructive sleep apnea [5,6,7]. The association of DS with metabolic syndrome (MetS) features dramatically increases the risk of developing the currently renamed metabolic dysfunction-associated steatotic liver disease (MASLD) [8]. Indeed, previous studies reported a predisposition to fatty liver in children with DS [9,10]. Valentini et al. [9] reported a 64.3% prevalence of hepatic steatosis, with severe steatosis present in approximately 4% of children with DS, demonstrating a more significant risk of MASLD compared to the general pediatric population. Interestingly, the same authors demonstrated that this trend was independent of the obese phenotype [10]. Conversely, the presence of steatosis in children with DS was associated with the presence of the well-known rs738409 variant in the gene encoding for patatin-like phospholipase domain-containing-3, and overall, this variant was significantly associated with the risk of severe steatosis.

No pharmacological therapies approved by the Food and Drug Administration are indicated for MASLD in the pediatric population [11]. This represents a substantial treatment gap, particularly in children with DS, in which advances in medical care have improved life expectancy, thus enhancing the long-term cardiometabolic issues that go along with obesity rising and MASLD progression. However, even though the preliminary results of several ongoing trials with pharmacological agents are promising in MASL, currently, intervention, including a balanced nutritional approach and physical exercise, remains the first-line recommended approach for pediatric and adult MASLD to promote hepato-metabolic improvement [11,12]. Despite the absence of specific diet recommendations, evidence from the literature highlights that both the low-sugar-free diet and the Mediterranean diet (MD) effectively reduce steatosis in children with MASLD [11,13]. In particular, MD is characterized by a high intake of whole grains, legumes, fruits, vegetables, and healthy fats, especially olive oil, which may have several biochemical and molecular effects on the cardiovascular system [14]. Moreover, due to its plant-based composition, MD also possesses antioxidant and anti-inflammatory properties. Overall, MD is designed to reduce the consumption of animal fats and cholesterol in a diet with an appropriate balance between energy intake and expenditure [15]. MD is widely accepted to play a crucial role in reducing body mass index (BMI), fat mass, hepatic steatosis, and insulin resistance, while also improving transaminase levels, with positive effects on inflammation and oxidative stress in children and adolescents [16,17,18,19].

In addition, several dietary supplements appear to be effective in improving MASLD-related clinical and histological features, despite the limited number of prospective clinical trials and the absence of standardized guidelines for their use [20,21]. Therefore, healthcare professionals follow an evidence-based approach to the use of supplements, such as vitamins and antioxidants, in combination therapy with lifestyle approaches in pediatric MASLD [22].

Currently, no data are available on the potential benefits of these non-pharmacological approaches in children with DS affected by MASLD. Hence, the present retrospective longitudinal study aims to evaluate the effects of nutritional counseling on MASLD and support pediatric patients in moving forward with a multidisciplinary approach based on adherence to the MD, regular physical activity, and natural supplements with antioxidant principles.

## 2. Materials and Methods

### 2.1. Study Design and Population

The present study is an observational study that retrospectively evaluated a cohort of 54 children with Down syndrome (DS) who were already affected by MASLD, and among them, 31 adhered to a six-month lifestyle intervention. [10]. All patients were advised to maintain regular physical activity, follow a dietary intervention based on MD, and take one antioxidant supplement orally. Patients were enrolled from the DS outpatient clinic of the Pediatric Department at the Bambino Gesù Children’s Hospital in Rome. The recruitment period was from January 2016 to December 2021.

According to the new definition, MASLD is characterized by the presence of hepatic steatosis (detected by imaging or biopsy), plus at least 1 of the following cardiometabolic criteria: (1) body mass index (BMI) ≥ 85th centile for age and sex or waist circumference > 95th centile; (2) fasting serum glucose ≥ 100 mg/dL or 2 h post-load glucose level ≥ 140 mg/dL or glycated hemoglobin (HbA1c) ≥ 5.7% or on specific drug treatment for type two diabetes; (3) blood pressure ≥ 130/80 mmHg for children aged <13 years or ≥130/85 mmHg for children aged ≥13 years or specific drug treatment for hypertension; (4) plasma triglycerides ≥ 100 mg/dL for children aged <10 years or ≥150 mg/dL for children aged ≥10 years; and (5) plasma HDL cholesterol ≤ 40 mg/dL or specific lipid lowering treatments [22].

All patients were advised to maintain regular physical activity, follow a dietary intervention based on MD, and take one antioxidant supplement orally.

Regarding physical activity, the counseling approach was mainly used to stimulate patients to increase their physical activity, taking into consideration the motor disability of the study population [23]. In particular, for the physical activity component, recommendations were aligned with current international guidelines for managing pediatric obesity. Children and adolescents were encouraged to engage in at least 60 min of moderate to vigorous physical activity per day, primarily of an aerobic nature (such as brisk walking, cycling, or active play). In addition, the intervention included guidance to incorporate muscle- and bone-strengthening activities (e.g., jumping, running, or resistance-based exercises) at least three times per week, as well as strategies to minimize sedentary behaviors, particularly screen time. These recommendations aimed to support sustainable, age-appropriate, and enjoyable physical activity habits within each child’s daily routine. Unfortunately, the historical period in which the study was conducted was marked by the Coronavirus disease 2019 (COVID-19) pandemic, during which sport and recreational activities were significantly limited [24].

With regard to the nutritional intervention, participants were advised to adopt a dietary pattern consistent with the Mediterranean diet, characterized by a high intake of vegetables, fruits, whole grains, legumes, and potatoes; moderate consumption of fish and poultry; and limited intake of full-fat dairy products, red and processed meats, and homemade sweets. To enhance adherence, the same dietary recommendations were extended to the entire family, promoting a supportive home environment and shared behavioral change.

The suggested antioxidant supplement, recommended at one capsule per day, contained 10.0 mg of DL-alpha-tocopheryl acetate (Vitamin E, VitE), and 7.5 mg of hydroxytyrosol (HXT) from a standardized olive extract, all conveyed in extra-virgin organic olive oil (FENÒLIA^®^ formulation, P&P Farma S.r.l., Turin, Italy). To evaluate compliance with the recommendations, patients and their families were monitored every two months through call interviews conducted by our clinicians.

Liver enzymes, metabolic and anthropometric parameters, cardiovascular risk indexes, and hepatic steatosis grading by ultrasound were assessed at the first counseling (T0) visit and after 6 months (T1). Supplementary Table S1 presents the characteristics of the population at T0, including gender, age, anthropometric, and laboratory parameters.

This study was approved by the Ethics Committee of Bambino Gesù Children’s Hospital and was conducted in accordance with the principles of the Declaration of Helsinki (protocol number: 880_OPBG_2015). Written informed consent was obtained from parents or legal guardians.

### 2.2. Anthropometric and Biochemical Parameters

Body weight, height, and waist circumference (WC) were measured with the patient wearing underwear and without shoes. Two measurements of each parameter were taken by the same physician, and then the average was calculated. BMI, defined as weight in kilograms divided by height in meters squared (kg/m^2^), was reported as age and sex-specific percentiles calculated from CDC growth charts for pediatric patients according to the American Academy of Pediatrics Statement for children with DS [25].

Systolic and diastolic blood pressure (SBP and DBP) were measured on the right arm by one physician only, using a standard sphygmomanometer; the average of three blood pressure values was reported.

Venous blood samples were collected in the morning after an overnight fast of at least 8 h. Serum levels of total cholesterol, high-density lipoprotein (HDL)-cholesterol, low-density lipoprotein (LDL)-cholesterol, triglycerides, alanine aminotransferase (ALT), aspartate aminotransferase (AST), gamma-glutamyl transferase (GGT), C-reactive protein (CRP), glucose, insulin levels, and HbA1c were measured in all patients by using standard laboratory procedures.

The AST/ALT ratio was also measured to distinguish MASLD from other metabolic liver diseases and/or alcohol consumption, with values < 1 indicating MASLD.

The homeostasis model assessment score [HOMA-IR = (fasting insulin (µU/mL)  ×  fasting glucose (mmol/L))/22.5)] was used for estimating insulin resistance; a cut-off value of >2.5 was considered as an index of insulin resistance [26].

Anthropometric and biochemical parameters were assessed at the beginning (T0) and after 6 months (T1).

### 2.3. Liver Ultrasound

To overcome the operator-dependent accuracy and reliability issues, a liver ultrasound (US) was performed by the same radiologist, who is well-experienced in performing and interpreting hepatic US by using the Acuson P-300 (Siemens, München, Germany) equipped with a linear (5–12 MHz) and convex probe (1–8 MHz). US was performed at the beginning (T0) and after 6 months (T1).

The degree of steatosis was graded as absent (0), mild (1), moderate (2), and severe (3). Briefly, normal liver/absent steatosis was defined as having a normal liver echo-texture; mild steatosis as a slight and diffuse increase in fine parenchymal echoes with normal visualization of diaphragm and portal vein borders; moderate steatosis as a moderate and diffuse increase in fine echoes with slightly impaired visualization of diaphragm and portal vein borders; and severe steatosis as fine echoes with poor or no visualization of diaphragm, portal vein borders, and posterior portion of the right lobe [27].

### 2.4. Assessment of the Adherence to MD, Supplement Intake, and Regular Physical Activity

All patients underwent nutritional counseling with a specialist in our outpatient clinic. The approach aimed to deliver healthy eating guidelines and tips to the study participants and their families, as well as basic information on portion size and dietary recommendations. Tailored goals were set for each individual patient, and strategies for achieving them were discussed during the counseling. The same features were evaluated after 6 months, and on this occasion, the adherence to dietary recommendations was assessed.

To evaluate adherence to MD, the Mediterranean Diet Quality Index for children and teenagers (KIDMED) questionnaire was used. The KIDMED questionnaire is a widely used survey instrument for the youth population between 2 and 24 years, based on the consumption of foods representative of the MD. The questionnaire consists of 16 questions with yes/no answers and examines the daily or weekly eating habits of various food groups, including fruits, vegetables, fish, fast food, legumes, pasta and cereals, dairy products, dry fruits, olive oil, sweets, and confectionery. In addition, there are some specific questions about breakfast: habitual meal consumption, the consumption of cereals, bread, or rusk cookies, the consumption of snacks or cookies, and the consumption of milk or dairy products (yogurt, etc.).

According to Serra-Majem et al. [28], each response was assigned a score of +1 if it reflected a dietary behavior like the MD, or −1 for eating behaviors that diverged from those typical MD patterns. The final KIDMED score (ranging from 0 to 12), obtained by adding and subtracting the values assigned to the individual questions, provided a measure of the adherence to the MD. A score of less than 3 points was classified as representing low adherence to the MD (score 1), a score between 4–7 points was representative of medium adherence to the MD (score 2), while a score above 8 points was representative of high adherence to MD (score 3).

A brief survey was proposed to assess adherence to supplement intake at 6 months of counseling for patients and their families. The survey consisted of a 10 min interview conducted by doctors at our center, aimed at collecting information relating to adherence to the suggested antioxidant supplement, based on HXT and VitE. Patients were divided into two groups: those who consumed the supplement for the entire 6 months and those who did not. Those who did not take the supplement for the whole period were asked to provide the reason, likely due to the occurrence of side effects, forgetfulness, poor palatability of the product, or difficulty in swallowing the capsule.

To assess the physical activity of each patient, the IPAQ-International Physical Activity Questionnaire was used. This measure assessed the intensity and duration of physical activity and sitting time that people engaged in as part of their daily lives, and it was used to estimate total physical activity in MET-min/week and time spent sitting [29]. The score for physical activity was reported as follows: score 1 for patients who are physically inactive (individuals’ physical activity levels less than 700 MET-min/week), score 2 for patients who are sufficiently active (individuals’ physical activity levels from 700 to 2519 MET-min/week), and score 3 for patients who are highly active (individuals’ physical activity levels of 2520 MET-min/week or more).

### 2.5. Analysis of Markers of Low-Grade Systemic Inflammation

The levels of lipopolysaccharide (LPS) and four relevant cytokines, used for evaluating low-grade systemic inflammation, were assessed in plasma samples using commercially available kits. Plasma LPS concentration was measured by a commercially available kit (Amebocyte Lysate LAL Chromogenic Endpoint Assay; Hycult Biotech, Uden, the Netherlands). Plasma levels of interleukin (IL)-6, IL-1β, IL-10, and tumor necrosis factor (TNF)-α were measured according to the manufacturer’s recommendations by an enzyme-linked immunosorbent assay (BioVendor, Heidelberg, Germany). All these biomarkers were assessed at the beginning (T0) and after 6 months (T1).

### 2.6. Statistical Analyses

Data are reported as numbers or percentages for categorical variables and as median values plus 25th–75th centile ranges for non-normally distributed continuous variables. A paired *t*-test or a Wilcoxon signed-rank test was used to test differences in anthropometric, laboratory parameters, and steatosis grade between the baseline (T0) and after 6 months (T1). Correlation analyses were tested using the Spearman correlation.

Statistical analysis was performed using GraphPad software Prism9 (GraphPad Prism Inc., San Diego, CA, USA). Values < 0.05 were considered statistically significant.

### 2.7. GenAI

In this manuscript, Google Gemini AI 2.0 Flash was used to perform portions of the graphical abstract.

## 3. Results

### 3.1. The Population’s Characteristics and Adherence to Nutritional Counseling

In a previous observational study, we demonstrated that 54 out of 84 Caucasian children with DS were affected by MASLD [9]. During a routine medical visit for clinical follow-up and nutritional counseling, the patients were informed of the possibility of participating in the present observational study. After the recruitment, 31 patients provided consent to the study and agreed to be followed over time. All 31 patients attended the two-month interview and the clinical re-evaluation after six months.

**Figure 1:**
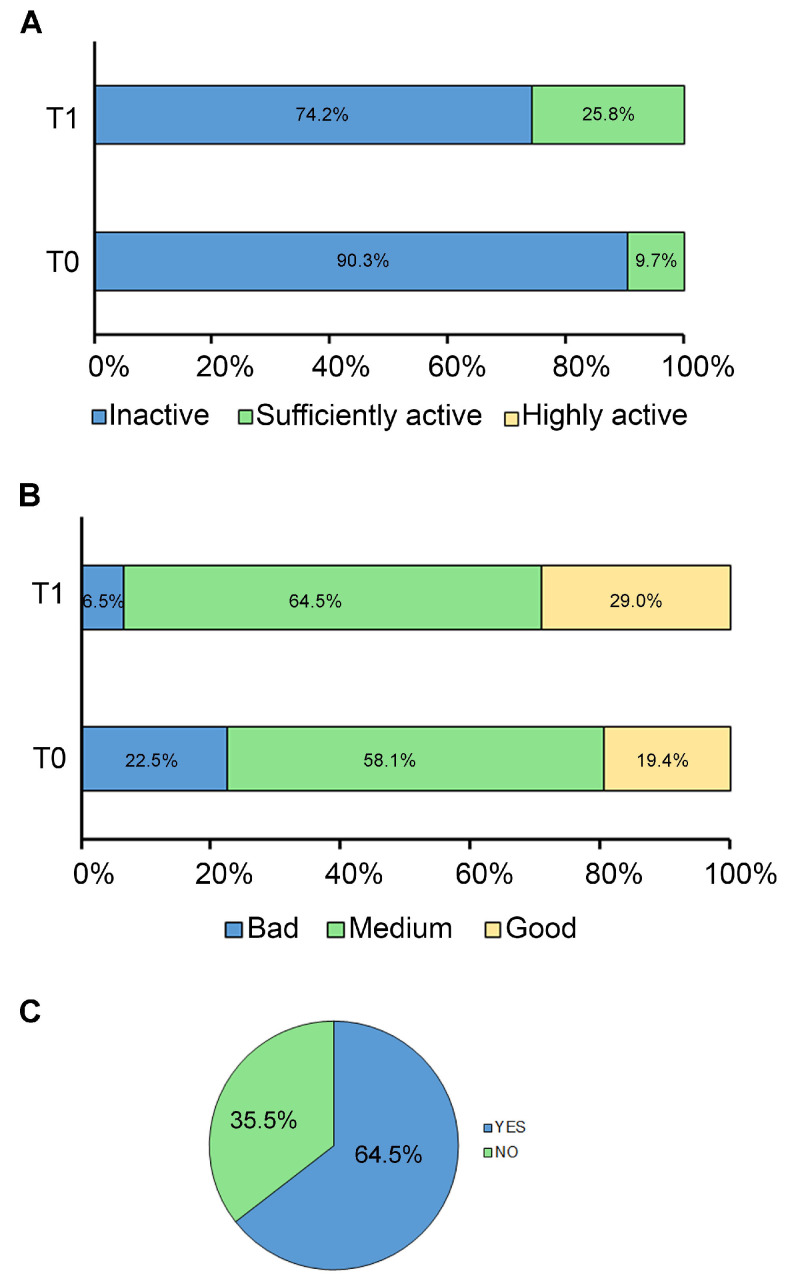
Adherence to lifestyle modifications. The graphs show the adherence of patients to (**A**) regular physical activity, (**B**) MD, and (**C**) HXT and VitE supplement at baseline (T0) and after 6 months (T1). Data are expressed as percentages.

According to the questionnaire results, the study confirmed that our population exhibited a high sedentary behavior at T0 while increasing its physical activity at T1. At the baseline (T0), almost the majority (90.30%) of patients were physically inactive, while only 3 (9.70%) were sufficiently active, and none were highly active. In contrast, after 6 months (T1), the percentage of patients who were physically inactive decreased to 74.2% (Figure 1A).

Adherence to the MD was poor in 7 (22.60%) patients, medium in 18 (58.10%) patients, and high or good in 6 (19.40%) patients at T0, while at T1, the percentages of medium and good adherence to MD increased to 64.5% and 29.0%, respectively (Figure 1B).

After the short survey, it was found that 20 patients (64.50%) took the oral dose of 7.5 mg of HXT and 10 mg of VitE for the 6-month follow-up period, while the remaining 11 patients (35.50%) did not (Figure 1C). No adverse events were reported by patients or their families. Of note, forgetfulness and difficulty swallowing the capsule were the most frequently reported issues for poor compliance with HXT supplementation.

Furthermore, as shown in Supplementary Table S2, we stratified patients according to their lifestyle patterns. Of note, 6 patients (19.35%) did not follow any nutritional or exercise recommendations, 5 patients (16.13%) followed the MD or exercise alone or in combination, and the remaining 20 patients (64.50%) consumed the suggested nutritional supplement daily for 6 months alone or in addition to the MD, and/or regular physical activity.

### 3.2. Impact of Lifestyle Changes on Anthropometric and Laboratory Parameters, Steatosis, and Low-Grade Systemic Inflammation

As reported in Table 1, after 6 months, patients exhibited statistically significant changes in anthropometrical and biochemical parameters, showing improvement in BMI (*p* = 0.02), BMI centile (*p* = 0.0004), WC (*p* = 0.0015), DBP (*p* = 0.0009), SBP (*p* = 0.0031), AST (*p* = 0.0095), fasting insulin (*p* = 0.03), and HOMA-IR (*p* = 0.02).

At the baseline, steatosis was severe in 6 (19.4%), moderate in 9 (29.0%), and mild in 16 (51.6%) patients. Table 2 describes the distribution of patients among steatosis severity at T0 and T1. After 6 months, steatosis regressed in 10 (31.0%) children with at least one grade improvement, and it was completely resolved in 2 (6.5%) patients.

Moreover, after 6 months, we observed a statistically significant reduction in plasma levels of the pro-inflammatory cytokine IL-1β (*p* = 0.0029) and a statistically significant increase in plasma levels of the anti-inflammatory cytokine IL-10 (*p* < 0.0001) (Table 3).

### 3.3. Impact of HXT + VitE Supplement Consumption on Steatosis, Anthropometric and Laboratory Parameters, Low-Grade Systemic Inflammatory Markers, and Steatosis After 6 Months

**Figure 2:**
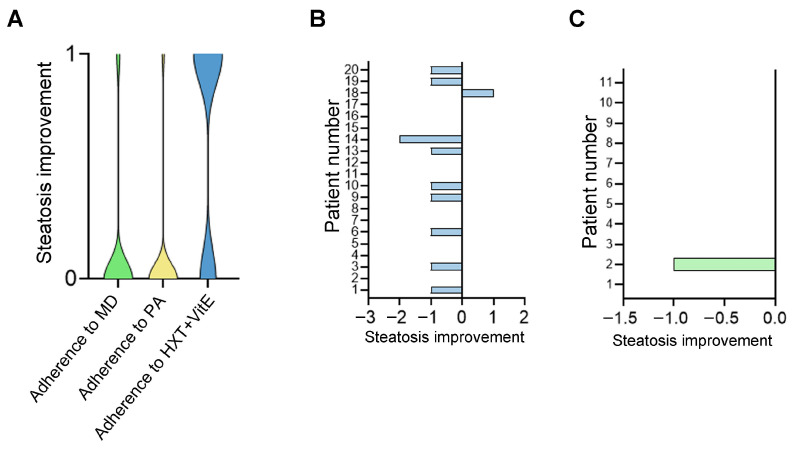
Regression of steatosis grade with different lifestyle modifications. The violin plot (**A**) illustrates the correlation between improvement in steatosis (at least 1 grade) and adherence to a medical diet (MD), regular physical activity (PA), or supplement consumption (HXT + VitE) following a 6-month follow-up. The histograms reported data in patients who did (**B**) and did not (**C**) consume HXT + VitE supplementation.

To evaluate the most efficient lifestyle modification leading to steatosis regression, we performed a correlation analysis, where the improvement in steatosis grade for each patient was associated with measures of adherence to MD, regular physical activity (PA), and HXT + VitE supplementation. As shown in Figure 2A, among the approaches proposed during counseling, the 6-month consumption of the natural supplement exhibited a better trend of correlation with steatosis regression (r = 0.31; *p* = 0.08) than adherence to MD (r = 0.02; *p* = 0.90) or regular physical activity (r = −0.15; *p* = 0.39).

Table 4 presents the impact of an HXT + VitE-based supplement on anthropometric and laboratory parameters, as well as low-grade chronic inflammation. In particular, WC, BMI calculated as kg/m^2^ and centile, DBP, SBP, fasting glucose, and levels of IL-1β were significantly (*p* < 0.05) decreased only in patients who regularly consumed the HXT and VitE-based supplement for the entire 6-month period. The circulating levels of IL-10 significantly improved in both patients who assumed and did not assume HXT + VitE (*p* < 0.0001). Moreover, although we observed a trend of a decrease in insulin and HOMA-IR values in both patient groups, the improvement was statistically significant only in the group that did not consume the supplement. When we compared the two groups after treatment (T1), we observed a significant difference in the AST/ALT ratio (Δ = −0.48), which was reduced in patients who regularly consumed HXT and VitE. A slight difference in HbA1c was also found between the two groups, even though the values in both groups remained under the pathological cut-off. Moreover, comparing the two groups at T1 we found a significant decrement in IL-1β levels (Δ = −140.6 pg/mL).

The percentage of patients with different steatosis grades who did or did not assume HXT + VitE is reported in Table 5. Out of the 20 patients who received HXT + VitE for 6 months, 11 (representing 55%) exhibited a regression of at least one grade in steatosis severity (Figure 2B). This observed improvement reached statistical significance (*p* < 0.0195). On the contrary, among the remaining 11 patients who did not receive HXT + VitE for the recommended period, only one showed an improvement in the steatosis grade (Figure 2C).

## 4. Discussion

The new MASLD definition highlights the importance of evaluating patients in a multidisciplinary setting, considering the various metabolic and hepatic phenotypes that characterize the disease [30]. Several pharmacological interventions have been proposed for the general population, but many trials have failed, while others are still ongoing [31]. Thus, lifestyle changes, including a balanced nutritional approach and regular physical exercise, remain the cornerstone of therapy for MASLD [32,33]. Combining physical activity, medications, and dietary interventions can be more effective than each prescription alone [34]. Recently, Zhang and Brandman [35] have highlighted the importance of lifestyle modification in the treatment of adults with MASLD. According to the American Association for the Study of Liver Diseases guidelines, reducing body weight and increasing physical exercise can lead to a 7% weight reduction, which has been shown to reverse steatohepatitis, and a 10% weight reduction can improve fibrosis [36]. In pediatric MASLD, limited data suggest that both aerobic and resistance exercise may be beneficial, producing small absolute reductions in hepatic steatosis and ALT levels. In particular, a randomized controlled trial (RCT) directly comparing 3 months of aerobic and resistance exercise three times per week in obese adolescent boys resulted in a modest (−1%) reduction in hepatic steatosis [37].

The best diet for pediatric age is the basis of many RCTs, but to date, systematic reviews on dietary lifestyle interventions for MASLD in children cite inconclusive results due to limitations in study design and small sample sizes. However, MD seems to be the most balanced for both pediatric and adult ages [36].

The MD has been proposed as the diet of choice for treating hepatic steatosis [38]. It can improve oxidative stress, inflammation, and insulin resistance, which are associated with intrahepatic lipid accumulation [14]. In this context, several clinical studies have been conducted to support the use of the MD in the treatment of MASLD. Della Corte et al. [19] investigated the effects of the MD on children with obesity and NAFLD. Specifically, children with poor adherence to the MD showed higher insulin levels during the oral glucose tolerance test (both at baseline and after 120 min) and increased HOMA-IR values, indicating impaired insulin sensitivity. Additionally, those with unhealthy eating habits had elevated CRP levels that reflected increased inflammation and fibrosis. Perez-Guisado et al. [39] confirmed the effect of the MD on the regression of steatosis degree and serologic improvement of liver function in a small Spanish study population. Overall, these findings corroborate current evidence on the liver-related outcomes of the MD [40,41,42,43].

Underlying the pathogenic mechanism of MASLD, in addition to insulin resistance, oxidative stress has been recognized as a key factor that may cause the depletion of antioxidant agents, resulting in an excess of oxygen-free radicals and the onset of hepatocyte damage [44,45]. Several studies conducted in the general population suggest that reducing oxidative stress may be a therapeutic strategy for MASLD [46]. In this regard, previous studies on pediatric cohorts demonstrated the efficacy of antioxidants (i.e., VitE) in blocking the development and progression of fibrosis and liver damage [13]. These molecules can significantly improve metabolic parameters, including oxidative stress, insulin resistance, visceral and liver fat, and transaminase levels [47]. To date, the results of these RCTs have been mainly negative or inconclusive, with no pharmacological breakthrough for children with MASLD. Five randomized, double-blind, placebo-controlled clinical trials have investigated the use of high-dose Vit E in children with NAFLD, with doses ranging from 600 IU/day to 800 IU/day [48]. However, VitE supplementation in pediatric MASLD was also combined with other supplements, such as docosahexaenoic acid [49]. Noteworthily, previous studies conducted in children with MASLD have demonstrated the efficacy of HXT, a phenol derived from olive oil with high antioxidant properties, and VitE in reducing the complications of obesity, such as oxidative stress, chronic inflammatory state, insulin resistance, and hepatic steatosis [50,51,52]. A study investigated the effect of HTX administration on weight and fat mass in women, and showed a statistically significant loss of weight and visceral fat mass (% weight loss: *p* = 0.012, % visceral fat loss: *p* = 0.006) in the group receiving the maximum HT dosage compared to a placebo after 4 weeks of intervention, as in our study, where treated patients achieved an improvement in BMI z-score as well as metabolic parameters [53]. Compared to the existing literature, even fewer studies have investigated the potential therapeutic effects of these natural compounds on the DS population. Some of them suggest polyphenol extracts as therapeutic interventions to prevent cognitive function impairment, given that mitochondrial dysfunction and oxidative stress are hallmarks contributing to the cognitive decline associated with DS [54,55,56,57].

To our knowledge, the present study was the first to evaluate the effectiveness of a multidisciplinary lifestyle approach, based on nutritional counseling and physical exercise, for treating patients with a diagnosis of MASLD and followed for DS. Notably, a regression of hepatic steatosis was observed in many patients after 6 months. The severity of steatosis decreased in a substantial number of patients, with some patients even resolving steatosis entirely (6.5%), particularly among those who took the recommended natural antioxidant supplementation. Moreover, MASLD was reduced not only by the degree of steatosis, as 6.5% no longer had steatosis, but as many as 11% resolved the picture of severe steatosis. In addition, a reduction in the mean values of cardiovascular factors (systolic blood pressure reduction of 6 mmHg and triglycerides reduction of 8%) and hepato-metabolic factors (ALT and HOMA-IR reduction) was observed in the group that also received the supplementation. Most of these effects could be linked to the antioxidant activity of HTX and VitE. Indeed, these two compounds, even if different in composition, act as direct radical-scavenging antioxidants modulating different down-stream target pathways, such as nuclear factor erythroid 2-related factor 2-dependent transcriptional regulation of genes that may reduce ROS reactive oxygen species and lipid peroxidation and improve mitochondrial homeostasis, thus decreasing hepato-metabolic impairment associated to MASLD [58,59,60].

Patients with DS have an increased risk of acquiring secondary metabolic pathologies primarily due to a physically inactive lifestyle and poor nutritional choices [61]. In line with that, most children of our cohort, at the first visit, were physically inactive and overweight or obese. However, after 6 months, the BMI decreased from a median of 28.93 to 27.98 kg/m^2^, and WC decreased from 93 to 87 cm. A fair percentage of patients (16.1%) improved their level of physical activity and their adherence to MD after 6 months of follow-up. The significant reduction of insulin resistance and AST also confirms the excellent results of our multidisciplinary lifestyle approach.

Finally, lifestyle changes were associated with a reduction in systemic inflammation markers. Specifically, after 6 months, the levels of the pro-inflammatory cytokine IL-1β decreased, and the levels of the anti-inflammatory cytokine IL-10 increased. A similar anti-inflammatory trend was observed in children with MASLD but without DS following lifestyle intervention and supplementation with HXT and VitE [52]. The anti-inflammatory effect of these two antioxidants was also confirmed in experimental models of MASLD where the reduction in systemic inflammation was explained with their activity on gut microbiota ecology (composition and amount) and beneficial effects on intestinal dysbiosis and barrier impairment that are pivotal mechanisms that sustain MASLD-related associated low-grade systemic inflammation in MASLD [62,63,64].

Despite the promising results, our study has certain limitations. First, hepatic steatosis was assessed by ultrasound, a non-invasive but operator-dependent and less sensitive and specific diagnostic tool, compared to liver biopsy or other imaging approaches. In this regard, it would also be interesting to evaluate liver fibrosis patterns using transient elastography to assess the impact of our lifestyle approach on liver damage, as previously reported by Panera et al. [52]. Given the pilot design of the study, sample size calculation was not conducted. Therefore, a second limitation is that our sample size may be too small to detect reliable effects, even if our results provide valuable preliminary findings that can inform future studies. A third limitation could be a selection bias, as socioeconomic status may have influenced recruitment, thus potentially affecting the representativeness of the sample. Indeed, adherence to the MD is known to be influenced by socioeconomic conditions, with a positive association between higher income and greater adherence. Similarly, the ability to engage in regular physical activity may depend on the availability of financial and logistical resources. Finally, it is important to note that the suggested nutritional supplement carries a cost, which may have further influenced participation. The fourth limitation of our study is that the cohort was evaluated at a single tertiary pediatric hospital in Italy. Therefore, further multi-center studies on larger cohorts, with different ethnicities, are recommended to overcome these limitations.

## 5. Conclusions

Our study is the first to report the efficacy of a comprehensive lifestyle intervention, based on a nutritional approach and physical exercise, on the severity of MASLD in children with DS. Moreover, our findings support the hypothesis that MD, a natural supplement with antioxidant properties (i.e., HXT), and regular exercise can be considered a safe therapeutic approach for reducing the risk and severity of MASLD, as well as the cardiometabolic risk, in children with DS. Overall, these findings support the choice of a non-pharmacological approach to mitigate metabolic and hepatic complications in this population.

Moreover, our study highlights that children with DS and MASLD need individualized physical activity programs that consider disability-related physical limitations, individualized dietary regimens, and personalized counseling designed to maximize treatment adherence and encourage self-empowerment.

## Figures and Tables

**Table 1:** Anthropometric and laboratory parameters at baseline (T0) and after 6 months (T1).

*Variables*	Median (25th–75th Centile) at T0	Median (25th–75th Centile) at T1	*p* Values
*Age*, years	11 (9.00 to 15.75)	12 (9.25 to 16.00)	-
*Sex* (Male)%	64.50%	64.50%	-
*Weight*, Kg	58.30 (38.85 to 73.95)	52.88 (42.50 to 71.95)	0.50
*BMI*, kg/m^2^	28.93 (24.25 to 33.70)	27.98 (23.91 to 31.77)	**0.02**
*BMI*, centile	91 (83.00 to 97.00)	90 (79.00 to 94.00)	**0.0004**
*WC*, cm (IQR)	93 (80.50 to 102.00)	87 (73.50 to 96.50)	**0.0015**
*DBP*, mmHg	72 (64.00 to 78.00)	58 (54.00 to 71.00)	**0.0009**
*SBP*, mmHg	124 (109.00 to 133.00)	108 (102.00 to 115.00)	**0.0031**
*Total cholesterol*, mg/dL	158 (136.00 to 182.00)	161 (138.00 to 168.00)	0.21
*HDL-cholesterol*, mg/dL	45 (41.00 to 54.00)	44 (40.00 to 51.00)	0.29
*LDL-cholesterol*, mg/dL	100 (83.00 to 118.00)	101 (78.00 to 113.00)	0.30
*Triglycerides*, mg/dL	88 (73.00 to 111.00)	80 (63.00 to 109.00)	0.24
*ALT*, UI/L	23 (17.00 to 36.00)	24 (17.00 to 30.00)	0.11
*AST*, UI/L	24 (20.00 to 28.00)	22 (18.00 to 26.00)	**0.0095**
*GGT*, UI/L	16 (13.00 to 19.00)	17 (13.00 to 23.00)	0.26
*CRP*, mg/dL	0.13 (0.06 to 0.50)	0.22 (0.09 to 0.50)	0.37
*Glucose*, mg/dL	90 (83.00 to 97.00)	88 (81.00 to 92.00)	0.08
*Insulin*, μUI/mL	20.75 (13.68 to 26.00)	16.5 (11.88 to 21.90)	**0.03**
*HbA1c*, mmol/mol	30 (28.00 to 34.00)	32 (30.00 to 34.00)	0.10
*HOMA-IR*	4.61 (3.00 to 5.64)	3.26 (2.59 to 4.93)	**0.02**
*ALT/AST ratio*	0.94 (0.79 to 1.43)	0.93 (0.73 to 1.29)	0.79

Values are expressed as percentages or median plus 25th–75th centile values for non-normally distributed continuous variables. *p* ≤ 0.05, significant difference was reported in bold. BMI, body mass index; WC, waist circumference; DBP, diastolic blood pressure; SBP, systolic blood pressure; HDL, high density lipoprotein; LDL, low density lipoprotein; ALT, alanine aminotransferase; AST aspartate aminotransferase; GGT, gamma-glutamyl transferase; CRP, C-reactive protein; HbA1c; glycated haemoglobin; HOMA-IR, homeostasis model assessment score.

**Table 2:** Steatosis grade at baseline (T0) and after 6 months (T1).

*Steatosis Grade*	Number of Patients	Percentage
***at T0***		
0	0	0.00%
1	16	51.60%
2	9	29.00%
3	6	19.40%
***at T1***		
0	2	6.50%
1	16	51.60%
2	13	41.90%
3	0	0.00%

**Table 3:** Markers of low-grade systemic inflammation at baseline (T0) and after 6 months (T1).

*Parameters*	Median (25th–75th Centile) at T0	Median (25th–75th Centile) at T1	*p* Values
*LPS*, EU/mL	8.9 (7.90 to 11.10)	9.10 (7.90 to 11.10)	0.83
*IL-6*, pg/mL	190.9 (125.3 to 322.8)	243.1 (147.8 to 744.7)	0.33
*TNF-α*, pg/mL	199.7 (121.1 to 310.1)	204.1 (104.3 to 296.8)	0.62
*IL-1β*, pg/mL	335.0 (191.7 to 581.7)	144.2 (40.60 to 623.2)	**0.0029**
*IL-10*, pg/mL	67.70 (52.10 to 81.50)	184.4 (176.9 to 203.1)	**<0.0001**

Values are expressed as medians. *p* ≤ 0.05, a significant difference reported in bold. LPS, lipopolysaccharide; IL, interleukin; and TNF, tumor necrosis factor.

**Table 4:** Impact of HXT + VitE supplement on anthropometric and laboratory parameters, and low-grade chronic inflammatory markers, at baseline (T0) and after 6 months (T1).

	Without HXT + VitE			With HXT + VitE			*p* Values
*Parameters*	T0	T1	*p* Values T0 vs. T1	T0	T1	*p* Values T0 vs. T1	T1 with vs. T1 Without HXT + VitE
*Age*, years	10 (8.25 to 12.50)	10 (8.25 to 12.75)		13 (11.00 to 16.50)	14 (11.50 to 17.00)	-	**-**
*Sex* (M)%	81.80	81.80		55.00	55.00	-	-
*Weight*, Kg	40.98 (33.40 to 73.95)	43.33 (34.60 to 70.00)	0.17	61.55 (44.89 to 75.91)	61.03 (45.46 to 73.76)	0.99	0.11
*BMI*, kg/m^2^	23.99 (22.73 to 32.60)	23.94 (22.66 to 30.44)	0.36	30.23 (25.48 to 33.79)	28.87 (26.10 to 33.57)	**0.04**	0.13
*BMI*, centile	89.00 (87.00 to 97.00)	88.00 (86.00 to 95.00)	0.07	91.50 (81.25 to 96.00)	90.00 (72.00 to 93.50)	**0.004**	0.21
*z-BMI*	1.97 (1.69 to 2.57)	1.88 (0.70 to 2.02)	0.82	2.18 (1.92 to 2.35)	1.86 (1.56 to 2.15)	0.81	0.13
*WC*, cm	81.50 (72.50 to 97.00)	76.00 (67.00 to 95.00)	0.06	94.50 (83.69 to 103.0)	92.88 (80.75 to 97.25)	**0.015**	0.08
*DBP*, mmHg	71.00 (54.00 to 75.00)	63.00 (54.00 to 75.00)	0.15	73.00 (64.25 to 82.13)	57.50 (54.00 to 61.25)	**0.002**	0.26
*SBP*, mmHg	115.0 (102.0 to 126.0)	107.0 (101.0 to 114.0)	**0.03**	127.0 (109.0 to 134.4)	110.5 (102.0 to 118.8)	**0.04**	0.23
*Total cholesterol*, mg/dL	166.0 (144.0 to 190.0)	161.0 (155.0 to 176.0)	0.83	158.0 (135.3 to 181.1)	150.5 (130.3 to 167.5)	0.19	0.33
*HDL-cholesterol*, mg/dL	46.00 (39.00 to 58.00)	49.00 (38.00 to 57.00)	0.45	44.00 (42.00 to 49.25)	43.50 (40.25 to 49.00)	0.49	0.5
*LDL-cholesterol*, mg/dL	104.0 (86.00 to 118.0)	106.0 (93.00 to 114.0)	0.84	97.50 (81.50 to 120.5)	92.50 (78.00 to 110.3)	0.17	0.49
*ALT*, UI/L	23.00 (17.00 to 40.00)	23.00 (14.00 to 29.00)	0.47	24.50 (17.25 to 35.50)	24.50 (17.25 to 34.50)	0.15	0.21
*AST*, UI/L	28.00 (20.00 to 34.00)	23.00 (21.00 to 30.00)	0.08	24.00 (19.75 to 26.00)	21.50 (17.00 to 25.00)	0.06	0.11
*GGT*, UI/L	16.00 (13.00 to 19.00)	19.00 (14.00 to 25.00)	0.25	15.50 (13.00 to 18.75)	16.5.0 (12.25 to 21.50)	0.84	0.17
*AST/ALT*	1.47 (0.95 to 1.68)	1.30 (1.04 to 1.50)	0.93	0.86 (0.75 to 1.13)	0.82 (0.66 to 1)	0.60	**0.001**
*CRP*, mg/dL	0.090 (0.050 to 0.29)	0.130 (0.050 to 0.240)	0.79	0.160 (0.070 to 0.590)	0.240 (0.110 to 0.590)	0.37	0.79
*Triglycerides*, mg/dL	89.00 (69.00 to 118.00)	80.00 (67.00 to 109.00)	0.53	86.50 (76.00 to 107.5)	77.00 (61.50 to 107.00)	0.22	0.56
*Glucose*, mg/dL	88.00 (86.00 to 98.00)	89.00 (83.00 to 93,00)	0.75	90.50 (82.00 to 96.25)	86.00 (79.50 to 91.50)	**0.05**	0.16
*HbA1c*, mmol/mol	28.00 (27.00 to 30.00)	30.00 (28.00 to 33.00)	0.77	31.50 (30.00 to 34.75)	32.00 (30.50 to 35.00)	0.45	**0.04**
*Insulin*, μUI/mL	20.40 (13.90 to 25.90)	14.30 (10.40 to 20.70)	**0.04**	23.00 (13.60 to 26.30)	17.00 (13.50 to 23.60)	0.27	0.27
*HOMA-IR*	4.272 (2.952 to 5.628)	2.860 (2.054 to 4.498)	**0.04**	4.657 (3.018 to 5.679)	3.442 (2.629 to 5.007)	0.24	0.49
*LPS*, EU/mL	9.700 (6.800 to 15.60)	8.800 (7.800 to 9.900)	0.51	8.850 (7.600 to 10.75)	9.300 (7.950 to 11.98)	0.43	0.31
*IL-6*, pg/mL	296.3 (176.6 to 367.2)	185.3 (136.9 to 351.9)	0.20	141.0 (107.3 to 240.7)	280.2 (150.1 to 929.0)	0.06	0.1
*TNF-α*, pg/mL	226.2 (131.1 to 295.9)	240.5 (193.8 to 321.6)	0.51	174.8 (111.9 to 316.7)	188.3 (97.03 to 239.4)	0.27	0.1
*IL-1β*, pg/mL	326.1 (191.7 to 531.1)	270.6 (72.20 to 914.2)	0.89	373.2 (145.7 to 655.4)	130.0 (29.18 to 285.9)	**0.0001**	**0.05**
*IL-10*, pg/mL	78.20 (62.60 to 91.10)	181.0 (171.9 to 191.3)	**0.0010**	66.95 (47.90 to 79.08)	185.3 (178.0 to 211.9)	**<0.0001**	0.32

Values are expressed as percentage or median plus 25th–75th centile values for non-normally distributed continuous variables. *p* ≤ 0.05, significant difference reported in bold. BMI, body mass index; WC, waist circumference; DBP, diastolic blood pressure; SBP, systolic blood pressure; HDL, high-density lipoprotein; LDL, low-density lipoprotein; ALT, alanine aminotransferase; AST aspartate aminotransferase; AST/ALT ratio: GGT, gamma-glutamyl transferase; CRP, C-reactive protein; HbA1c; glycated hemoglobin; HOMA-IR, homeostasis model assessment score; LPS, lipopolysaccharide; IL, interleukin; and TNF, tumor necrosis factor.

**Table 5:** Improvement in steatosis with respect to HXT and VitE intake.

*Steatosis Grade*	*Without HXT + VitE*	*With HXT + VitE*
***at T0***		
0	0 (0.00%)	0 (0.00%)
1	8 (72.70%)	8 (40.00%)
2	2 (18.20%)	7 (35.00%)
3	1 (9.10%)	5 (25.00%)
***at T1***		
0	0 (0.00%)	0 (0.00%)
1	1 (9.10%)	14 (70.00%)
2	6 (54.50%)	6 (30.00%)
3	4 (36.40%)	0 (0.00%)

## Data Availability

The data are unavailable due to privacy restrictions.

## Supplementary Materials

The following supporting information can be downloaded at https://www.mdpi.com/article/10.3390/nu17142331/s1, Table S1: Anthropometric and laboratory characteristics of study population; Table S2: Stratification of patients accordingly to lifestyle patterns of patients at baseline (T0) and after 6 months (T1).

## Abbreviations

The following abbreviations are used in this manuscript:

MASLD	metabolic dysfunction-associated steatotic liver disease
DS	Down syndrome
MetS	metabolic syndrome
MD	Mediterranean diet
BMI	body mass index
VitE	vitamin E
HXT	hydroxytyrosol
WC	waist circumference
DBP	diastolic blood pressure
SBP	systolic blood pressure
HDL	high-density lipoprotein
LDL	low-density lipoprotein
ALT	alanine aminotransferase
AST	aspartate aminotransferase
GGT	gamma-glutamyl transferase
CRP	C-reactive protein
HbA1c	glycated hemoglobin
HOMA-IR	homeostasis model assessment score
KIDMED	mediterranean diet quality index
IPA-Q	international physical activity questionnaire
LPS	lipopolysaccharide
IL	interleukin
TNF	tumor necrosis factor
