# Supplementary material for: Lifestyle Intervention Improves Metabolic Dysfunction-Associated Steatotic Liver Disease in Children with Down Syndrome
Source: Nutrients. 2025 Jul 16;17(14):2331. doi: 10.3390/nu17142331 (PMC12299393; doi:10.3390/nu17142331)
Supplement: Supplementary file 1 [file nutrients-17-02331-s001.zip › nutrients-3720654-supplementary.pdf]

Supplementary Data

Lifestyle Intervention Improves Metabolic Dysfunction-Associated Steatotic Liver Disease in Children with Down Syndrome

Vittorio Scoppola, Annalisa Crudele, Antonella Mosca, Nadia Panera, Chiara di Camillo, Caterina Bock, Massimiliano Raponi, Alberto Villani, Anna Alisi and Diletta Valentini

Table S1. Anthropometric and laboratory characteristics of study population.

Variables	**Median (25th-75th centile)**
Age, years	11 (9.00 to 15.75)
Sex (M) %	64.50%
Weight, Kg	58.3 (39.14 to 73.81)
BMI, kg/m²	28.93 (24.26 to 33.62)
BMI, centile	91 (83.50 to 96.75)
WC, cm	93 (80.62 to 102.00)
DBP, mmHg	72 (64.25 to 77.75)
SBP, mmHg	124 (109.00 to 132.50)
Total cholesterol, mg/dL	158 (136.75 to 181.50)
HDL-cholesterol, mg/dL	45 (41.25 to 53.00)
LDL-cholesterol, mg/dL	100 (83.25 to 117.00)
Triglycerides, mg/dL	88 (73.75 to 110.25)
ALT, U/L	23 (17.00 to 35.50)
AST, U/L	24 (20.50 to 28.00)
GGT, U/L	16 (13.00 to 19.00)
CRP, mg/dL	0.135 (0.06 to 0.48)
Glucose, mg/dL	90 (83.25 to 96.50)
Insulin, µU/mL	20.75 (13.70 to 25.90)
HbA1C, mmol/mol	30 (28.25 to 34.00)
HOMA-IR	4.615 (3.01 to 5.62)

Values are expressed as a percentage or median. BMI, body mass index; WC, waist circumference; DBP, diastolic blood pressure; SBP, systolic blood pressure; HDL, high density lipoprotein; LDL, low density lipoprotein; ALT, alanine aminotransferase; AST aspartate aminotransferase; GGT, gamma-glutamyl transferase; CRP, C-reactive protein; HbA1c; glycated hemoglobin; HOMA-IR, homeostasis model assessment score.

Table S2. Stratification of patients accordingly to lifestyle patterns of patients at baseline (T0) and after 6 months (T1).

KIDMED score at T0 (1 = low adherence, 2 = medium adherence; 3 = high adherence)	Score exercise at T0 (1 = physically inactive; 2 = sufficiently active)	HXT+VitE consumption at T0 (0 = NO; 1 = YES)	KIDMED score at T1 (1= low adherence, 2= medium adherence; 3= high adherence)	Score exercise at T1 (1 = physically inactive; 2 = sufficiently active)	HXT+VitE consumption at T1 (0 = NO; 1 = YES)	Lifestyle patterns
2	1	0	2	1	0	-
1	1	0	1	1	0	-
2	1	0	2	1	0	-
2	1	0	2	1	0	-
3	1	0	3	1	0	-
2	1	0	2	1	0	-
3	1	0	2	2	0	E
2	1	0	3	1	0	MD
2	1	0	3	1	0	MD
1	1	0	2	1	0	MD
2	1	0	3	2	0	MDE
1	1	0	1	1	1	S
2	1	0	2	1	1	S
2	1	0	2	1	1	S
3	2	0	3	1	1	S
2	1	0	2	1	1	S
3	2	0	3	1	1	S
3	1	0	2	1	1	S
2	2	0	2	2	1	S
2	1	0	2	1	1	S
2	1	0	2	2	1	SE
2	1	0	2	2	1	SE
3	1	0	2	2	1	SE
2	1	0	2	2	1	SE
2	1	0	3	1	1	SMD
1	1	0	2	1	1	SMD
1	1	0	2	1	1	SMD
1	1	0	2	1	1	SMD
2	1	0	3	1	1	SMD
2	1	0	3	1	1	SMD
1	1	0	2	2	1	SMDE

S, supplement only; E, physical exercise only; MD, mediterranean diet only; SMD, supplement and mediterranean diet; SE, supplement and physical exercise; SMDE, supplement, mediterranean diet and physical exercise; MDE, mediterranean diet and physical exercise; blank line, no of the above: blank line; HXT, hydroxytyrosol; VitE, vitamin E.
